# Transcriptomic analyses provide molecular insight into the cold stress response of cold-tolerant alfalfa

**DOI:** 10.1186/s12870-024-05136-y

**Published:** 2024-08-03

**Authors:** Xiaojian Pu, Yunjie Fu, Chengti Xu, Xiuzhang Li, Wei Wang, Kejia De, Xijie Wei, Xixi Yao

**Affiliations:** 1 Academy of Animal Husbandry and Veterinary Science, Qinghai University, No.1 Wei’er Road, Biopark, Chengbei District, Xining, Qinghai 810016 China; 2 College of Agriculture and Animal Husbandry, Qinghai University, Xining, 810016 Qinghai Province China

**Keywords:** Alfalfa, Cold stress, Metabolic pathways, Transcription factor, Plant hormones

## Abstract

**Background:**

Daye No.3 is a novel cultivar of alfalfa (*Medicago sativa* L.) that is well suited for cultivation in high-altitude regions such as the Qinghai‒Tibet Plateau owing to its high yield and notable cold resistance. However, the limited availability of transcriptomic information has hindered our investigation into the potential mechanisms of cold tolerance in this cultivar. Consequently, we conducted *de novo* transcriptome assembly to overcome this limitation. Subsequently, we compared the patterns of gene expression in Daye No. 3 during cold acclimatization and exposure to cold stress at various time points.

**Results:**

A total of 15 alfalfa samples were included in the transcriptome assembly, resulting in 141.97 Gb of clean bases. A total of 441 DEGs were induced by cold acclimation, while 4525, 5016, and 8056 DEGs were identified at 12 h, 24 h, and 36 h after prolonged cold stress at 4 °C, respectively. The consistency between the RT‒qPCR and transcriptome data confirmed the accuracy and reliability of the transcriptomic data. KEGG enrichment analysis revealed that many genes related to photosynthesis were enriched under cold stress. STEM analysis demonstrated that genes involved in nitrogen metabolism and the TCA cycle were consistently upregulated under cold stress, while genes associated with photosynthesis, particularly antenna protein genes, were downregulated. PPI network analysis revealed that ubiquitination-related ribosomal proteins act as hub genes in response to cold stress. Additionally, the plant hormone signaling pathway was activated under cold stress, suggesting its vital role in the cold stress response of alfalfa.

**Conclusions:**

Ubiquitination-related ribosomal proteins induced by cold acclimation play a crucial role in early cold signal transduction. As hub genes, these ubiquitination-related ribosomal proteins regulate a multitude of downstream genes in response to cold stress. The upregulation of genes related to nitrogen metabolism and the TCA cycle and the activation of the plant hormone signaling pathway contribute to the enhanced cold tolerance of alfalfa.

**Supplementary Information:**

The online version contains supplementary material available at 10.1186/s12870-024-05136-y.

## Background

Alfalfa (*Medicago sativa* L.) is recognized as an important forage crop on a global scale [1]. The rapid growth of the animal husbandry industry in China has led to a significant increase in the demand for alfalfa. Nevertheless, insufficient domestic production capacity has led to a heavy dependence on imports to meet demand. Regrettably, the average self-sufficiency rate for high-quality alfalfa was shown to be as low as 64% [2]. Extremely cold climates are the primary factor restricting alfalfa yields [3–5]. The overwintering survival rate and fresh yield of alfalfa decrease significantly in high-altitude regions of the Qinghai‒Tibet Plateau due to the low temperatures [6]. Feng et al. [2] reported a significant positive correlation between alfalfa yield and environmental temperature. Therefore, the cultivation and acclimation of cold-tolerant alfalfa, as well as understanding the molecular mechanisms underlying its response to cold stress, are highly important for breeding new varieties and for the development of the livestock industry.

Cold stress is commonly known to impede plant growth, induce leaf yellowing, and result in the build-up of reactive oxygen species, along with membrane damage [7]. During cold stress, the chloroplast is typically the organelle that is most significantly and rapidly impacted among all cell structures [8]. Low temperatures can inhibit electron transport through changes in the physical properties of thylakoid lipids, leading to increased membrane viscosity. Additionally, they exert a stronger negative effect on enzymatic reactions involved in C, N, and S reduction than on photophysical and photochemical processes related to light absorption, energy transfer, and transformation [9]. Photoinhibition is reversible and occurs due to increased thermal dissipation of energy, resulting in the downregulation of PSII activity and a decrease in effective PSII [10]. Ribosomal proteins were reported to be correlated with photosynthesis under cold stress. In an *Arabidopsis* mutant lacking the ribosomal protein S5, the expression of genes associated with photosynthesis and the cold stress response was inhibited [11]. Wang et al. [12] showed that the rice (*Oryza sativa* L.) TCD11 gene encodes the ribosomal small subunit protein S6 and plays a crucial role in chloroplast development under low-temperature conditions. The induction of ribosomal protein genes can enhance translation processes and support the optimal functioning of ribosomes under low-temperature conditions [13].

Plant hormones have been widely reported to be involved in the response to cold stress. Under cold stress, abscisic acid (ABA) signaling has been found to regulate various transcription factors and induce the enrichment of genes associated with secondary metabolism, amino acid metabolism, carbohydrate metabolism, and lipid metabolism pathways, as reported in numerous studies [14]. An et al. [15] demonstrated that ABA insensitive 4 serves as an integrator of both jasmonic acid (JA) and ABA signals, allowing precise modulation of cold tolerance in apple (*Malus hupehensis*) through the regulation of APETALA2/ethylene responsive factor (AP2/ERF) and jasmonate-ZIM domain (JAZ) proteins. ABA signaling has also been linked to the brassinosteroid (BR) signaling pathway. The TOPLESS (TPL)/histone deacetylase 19 (HDA19) complex ensures an epigenetic connection between BR and ABA signaling through interactions among brassinazole-resistant 1 protein(BZR1)/BRI1-EMS-supressor 1 (BES1)-ABI3-ABI5 [16]. Therefore, ABA signaling is the key factor in the plant response to cold stress.

In alfalfa, pyrabactin resistance/pyr1-like (PYL) is strongly induced by cold stress [17]. Liu et al. [18] showed that receptor-like kinases, ribosomal proteins, protein synthesis and degradation genes are involved in controlling the dormancy of alfalfa under low temperature. The NAC family of transcription factors was also reported to play a potential role in enhancing the cold tolerance of alfalfa [19]. Overexpression of alfalfa calmodulin-like protein 46 (CML46) in tobacco enhances its tolerance to low temperatures [20]. Although these studies have identified numerous candidate genes involved in the enhancement of cold tolerance in alfalfa, the molecular mechanisms governing cold tolerance in alfalfa remain largely unexplored. In this study, we examined an alfalfa variety known as “Daye NO.3”. This variety has a winter survival rate of 90% at an altitude of 4,270 m on the Qinghai‒Tibet Plateau and achieves an annual fresh grass yield of more than 21 t/hm^2^ [21]. Our study analyzed the transcriptome expression profile of Daye NO.3 under low-temperature stress to (1) uncover the principal metabolic pathways involved in the response of Daye NO.3 to cold stress; (2) identify hub genes involved in the response to cold stress; and (3) explore the response of Daye NO.3 to cold stress in relation to plant hormone signal transduction.

## Methods

### Seedling cultivation and cold treatment

The experimental materials chosen for this study were the cold-tolerant cultivar ‘Daye NO.3 (D)’ and the cold-sensitive cultivar ‘LongDong (L)’ of alfalfa (*Medicago sativa* L.). The seeds were provided by the College of Animal Science and Veterinary Science, Qinghai University. Approximately 15 alfalfa seeds were evenly sown in a seedling bowl measuring 15 cm in diameter and 14 cm in height. The growth medium used was nutrient-rich soil. Following germination, the plant density was reduced to nine plants per pot, after which cultivation continued. The seedlings were cultured in a constant-temperature incubator, subjected to a 16-h light and 8-h dark cycle, with a light period at 23 °C and a dark period at 18 °C and a light intensity of 6000 lx.

After 18 days of seedling cultivation, cold treatment was carried out. The precultivated D and L seedlings were divided into two groups: a control group (CK) and a treatment group (T). The CK group was subjected to continued cultivation under the previous conditions, while the T group experienced a decrease in temperature to 4 °C for 12 h in the constant-temperature incubator, followed by a return to 23 °C. After three cycles of cold treatment, the temperature of the plants was maintained at 4 °C to induce cold stress.

### Determination of growth, photosynthetic pigment content, antioxidant enzyme activities, and malondialdehyde (MDA), H_2_O_2_, and O_2_^·^^−^contents

Following a 45-day period of cold stress, multiple physiological indicators were measured, and photographs of the phenotypes were obtained. The measurements and calculations of photosynthetic pigment content were performed following the methods described by [22]. The determination of peroxidase (POD), catalase (CAT), and superoxide dismutase (SOD) activities was conducted using the corresponding assay kits (BC0090 for POD, BC4780 for CAT, and BC5160 for SOD) from Beijing Solarbio Science & Technology Co., Ltd. (Beijing, China). The MDA, H_2_O_2_, and O_2_^·−^ contents were determined using the corresponding assay kits (BC0025 for MDA, BC3590 for H_2_O_2_, and BC1290 for O_2_^·−^) from Beijing Solarbio Science & Technology Co., Ltd. (Beijing, China). One-way analysis of variance (ANOVA) was employed to perform significance analysis of the relevant indicators among different treatment groups. Statistical significance was considered present for intergroup comparisons when *P* < 0.05. ANOVA was conducted using IBM SPSS Statistics software (Version 26.0, https://spss.mairuan.com/), and GraphPad Prism software (Version 9.0.0, https://www.graphpad.com/) was utilized for data visualization.

### RNA extraction, library construction, sequencing, and quality control

The aboveground parts of the D genotype plants were collected for RNA-Seq at T_0h (after cold acclimation), T_12h, T_24h, and T_36h (0 h, 12 h, 24 h, and 36 h after the temperature was maintained at 4 °C). Samples (0.2 g) from three biological replicates of each treatment were ground with liquid nitrogen for RNA extraction, and subsequently, the RNA samples were subjected to rigorous quality control measures. For accurate assessment of RNA integrity, an Agilent 2100 Bioanalyzer was used for quality control [23]. The library construction process was performed following the protocol described by Niu and Ma [24]. Initially, total RNA was randomly fragmented and utilized for the synthesis of first-strand cDNA using the M-MuLV reverse transcriptase system. Subsequently, the second strand of cDNA was synthesized employing DNA polymerase I and dNTPs. Then, AMPure XP beads were utilized to select cDNA fragments in the range of approximately 370–420 bp. The selected fragments were subjected to PCR amplification and purification using AMPure XP beads, ultimately yielding the library.

The cDNA library was sequenced using the Illumina NovaSeq platform, yielding raw sequencing data. To ensure the quality and reliability of the data analysis, filtering of the raw data was necessary. The removal of reads with adapters, reads containing N, and low-quality reads yielded clean sequencing data. Calculations of the Q20, Q30, and GC content were performed for the clean sequencing data. All subsequent analyses were conducted based on the analysis of the clean sequencing data from previous steps.

### Annotation of sequences and prediction of novel transcripts

The sequences within the clean data were aligned to the reference genome [1] of alfalfa utilizing HISAT2 as the alignment tool [25]. StringTie (version 1.3.3b) software was used for the prediction of the novel transcripts [26].

### Quantification, differential expression analysis, and enrichment analysis of genes

Gene quantification was performed using Feature Counts software (version 1.5.0-p3, http://www.bioconductor.org), and gene expression levels are reported as fragments per kilobase of exon model per million mapped fragments (FPKM) values. Differential expression analysis was conducted by comparing two groups using the R package DESeq2 software (version 1.20.0, https://www.bioconductor.org/packages/release/BiocViews.html#___Software). Genes demonstrating an absolute log2-fold change ≥ 2 and padj ≤ 0.05 were categorized as differentially expressed genes (DEGs). To explore the functions and metabolic pathways associated with the DEGs, they were annotated via the Kyoto Encyclopedia of Genes and Genomes (KEGG) and Gene Ontology (GO) databases, as described by Kanehisa and Goto [27] and the Gene Ontology Consortium [28].

### Protein‒protein interactions (PPIs) network analysis of DEGs

The analysis of protein‒protein interactions (PPIs) among DEGs was performed by using the STRING database (https://www.string-db.org/), which encompasses both known and predicted interactions. The interaction file of *Medicago truncatula* in the STRING database were used to predict the possible interaction. The protein sequence of DEGs were extracted through the TBtools software (Version 2.083) [29] and further searched in the STRING database through the “Single / Multiple Proteins by Sequence” function. The interaction relationships were further downloaded and visualized using the Cytoscape software (Version 3.8.2, https://cytoscape.org/).

### Short Time-series Expression Miner (STEM) analysis of genes

Genes were subjected to Short Time-series Expression Miner (STEM) analysis using their FPKM values [30]. First, the standard deviation of each gene across different treatment groups was calculated. Next, the genes were sorted in descending order based on their standard deviation. The top 5000 ranked genes were then subjected to STEM analysis. Genes exhibiting sustained increases or decreases in expression following stress treatment were subsequently annotated and analyzed for their functions in the KEGG database.

### Real-time quantitative polymerase chain reaction (RT‒qPCR)

Fresh alfalfa samples (0.2 g) were used for total RNA extraction, with three biological replicates of each treatment. Total RNA extraction was performed using the TianGen Total RNA Extraction Kit (DP419, TianGen, China), while cDNA library construction was performed with the PrimeScript™ RT Reagent Kit with gDNA Eraser (RR047A, TaKaRa, Japan). Gene sequences were extracted according to the transcriptome sequencing database, and primer design was conducted using the NCBI online primer design tool (https://www.ncbi.nlm.nih.gov/tools/primer-blast/). RT‒qPCR was carried out using 2x Universal Blue SYBR Green qPCR Master Mix (G3328-01, Servicebio, China). The reaction volume was 20 µL, and the reaction program was set according to the instructions provided with the kit. *Actin* was used as a reference gene to calculate the relative expression levels of selected genes [31].

## Results

### Comparison of phenotypes, photosynthetic pigments, and antioxidant capacity between the cold-tolerant genotype D and cold-sensitive genotype L

**Fig. 1:**
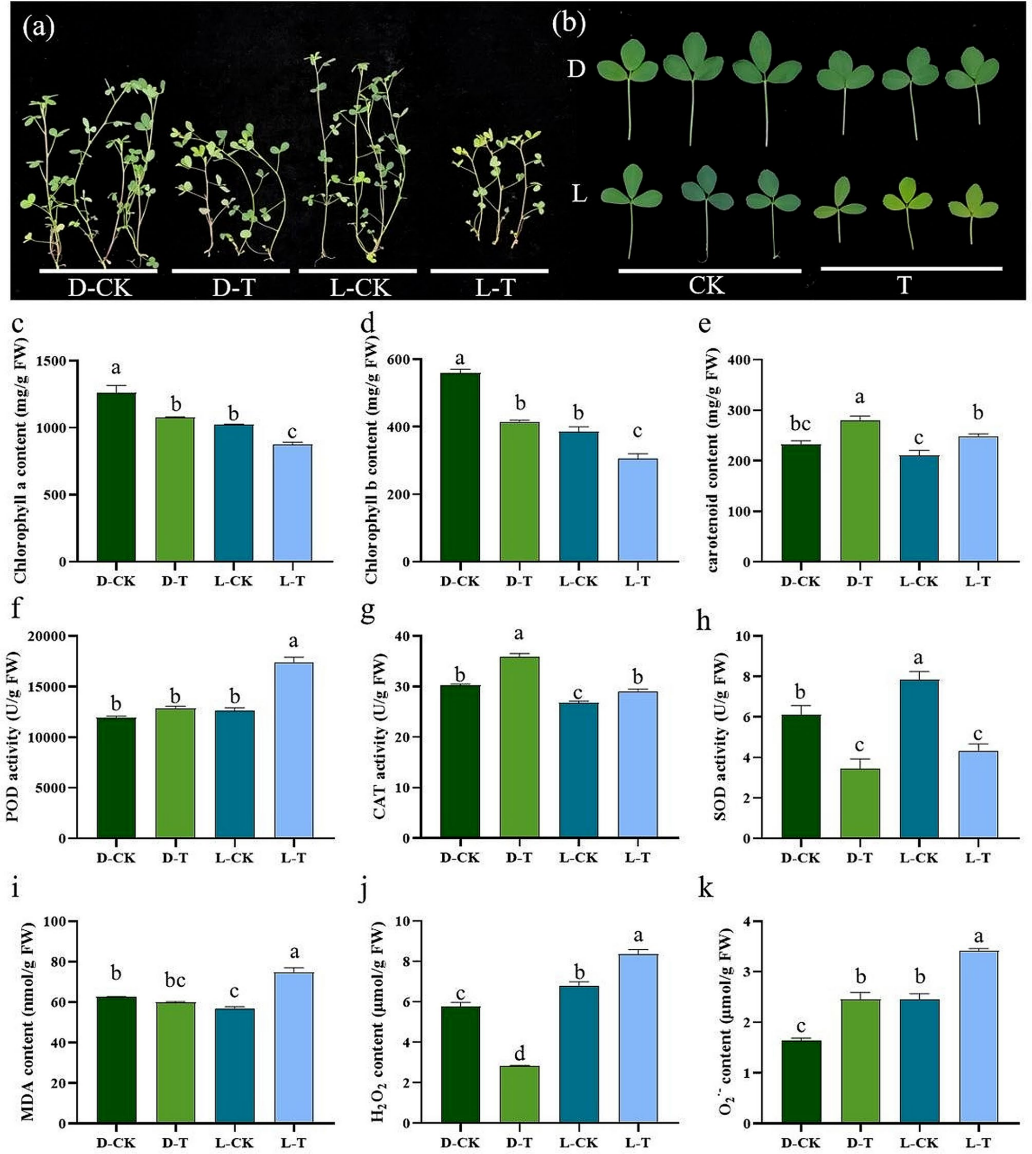
Effects of cold stress on growth (**a** and **b**), photosynthetic pigments (**c**–**e**), antioxidant enzyme activities (**f**–**h**), and reactive oxygen species metabolites (I–K) in the cold-tolerant genotype D and cold-sensitive genotype L; CK: control group; T: 4 °C cold stress treatment. Multiple comparisons were performed for each group using one-way analysis of variance (ANOVA). Different letters indicate significant differences (*P* < 0.05). Each group consisted of three biological replicates. The data are presented as the means ± SEs

The aboveground biomass of the D genotype was greater under cold stress than that of the L genotype (Fig. 1a). The leaves of the D genotype plants maintained their green color under cold stress, whereas those of the L genotype plants displayed pronounced yellowing (Fig. 1b). By measuring the content of photosynthetic pigments, it was found that under cold stress, both the D and L genotypes exhibited significant decreases in chlorophyll a and chlorophyll b compared to the control group (Fig. 1c and d). Furthermore, under stress conditions, the D genotype displayed significantly greater chlorophyll a, chlorophyll b, and carotenoid contents than did the L genotype (Fig. 1c and e). Through the analysis of antioxidant enzyme activities, it was observed that under cold stress, the L genotype exhibited a greater increase in POD activity, while the D genotype showed a greater increase in CAT activity (Fig. 1f and g). However, the activities of SOD were suppressed in both the D and L varieties under cold stress (Fig. 1h). Under cold stress, the MDA content in the D genotype did not significantly change compared to that in the CK (Fig. 1i). In contrast, the MDA content in the L genotype increased significantly (Fig. 1i). Moreover, the L genotype displayed significantly greater levels of H_2_O_2_ and O_2_^·−^ under cold stress, indicating that more severe oxidative damage was experienced by the L genotype than by the D genotype (Fig. 1j and k).

### Raw RNA-seq data and differential gene expression analysis

As shown in Table S1, transcriptome sequencing was carried out on 15 samples from both the control and treatment groups, yielding a combined total of 145.15 Gb of raw data. After the data were cleaned, a total of 141.97 Gb of clean bases were obtained. Each sample had more than 8 Gb of clean bases, while maintaining an RNA-seq error rate less than 0.3%. All the samples exhibited Q20 values exceeding 97%, with Q30 values exceeding 94%. The GC content was consistently maintained at approximately 41%. The findings of this study provide evidence that high-quality RNA-seq data are suitable for further analysis (Table S2).

**Fig. 2:**
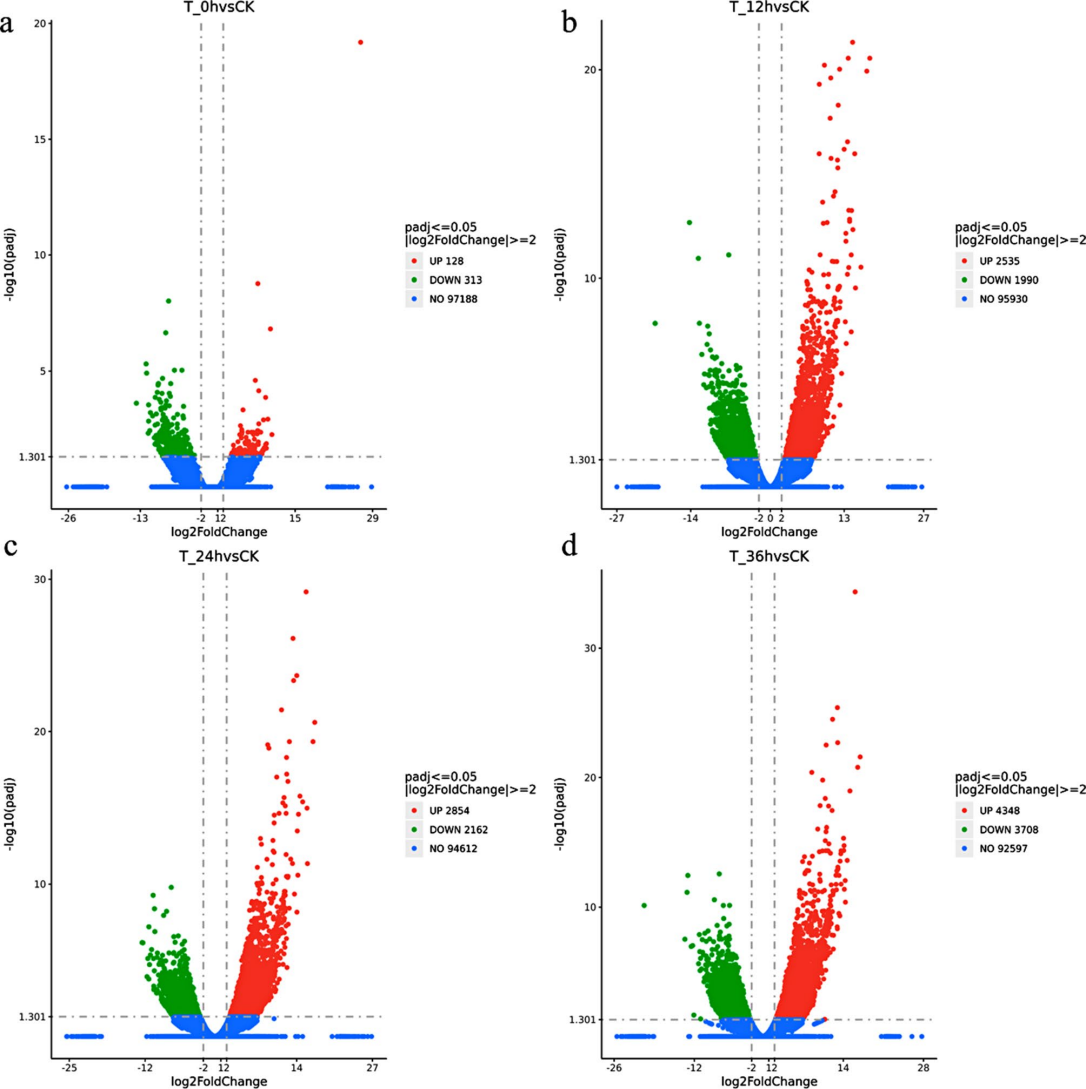
Cold-induced DEGs in alfalfa. The red dots represent upregulated DEGs, the green dots represent downregulated DEGs, and the blue dots represent non-DEGs. The position of a dot on the X-axis represents the expression level of the gene, while the position on the Y-axis represents the -log10(padj) value. CK stands for the control group, and T_0h, T_12h, T_24h, and T_36h represent 0 h, 12 h, 24 h, and 36 h of cold stress, respectively

After cold acclimation, differential expression analysis revealed 128 upregulated genes and 313 downregulated genes (Fig. 2a). These genes participate in the initial response to cold stress in alfalfa. After the samples were subjected to 12 h of cold stress treatment, 2535 genes were upregulated, and 1990 genes were downregulated, indicating that an increased number of genes responded to cold stress (Fig. 2b). The number of upregulated and downregulated genes continued to increase after 24 and 36 h of stress exposure (Fig. 2c and d). These findings indicate that as the duration of stress increases within the 0–36 h timeframe, there is a corresponding increase in the number of genes responding to cold stress.

### KEGG and GO enrichment analysis of DEGs involved in the cold response of alfalfa

**Fig. 3:**
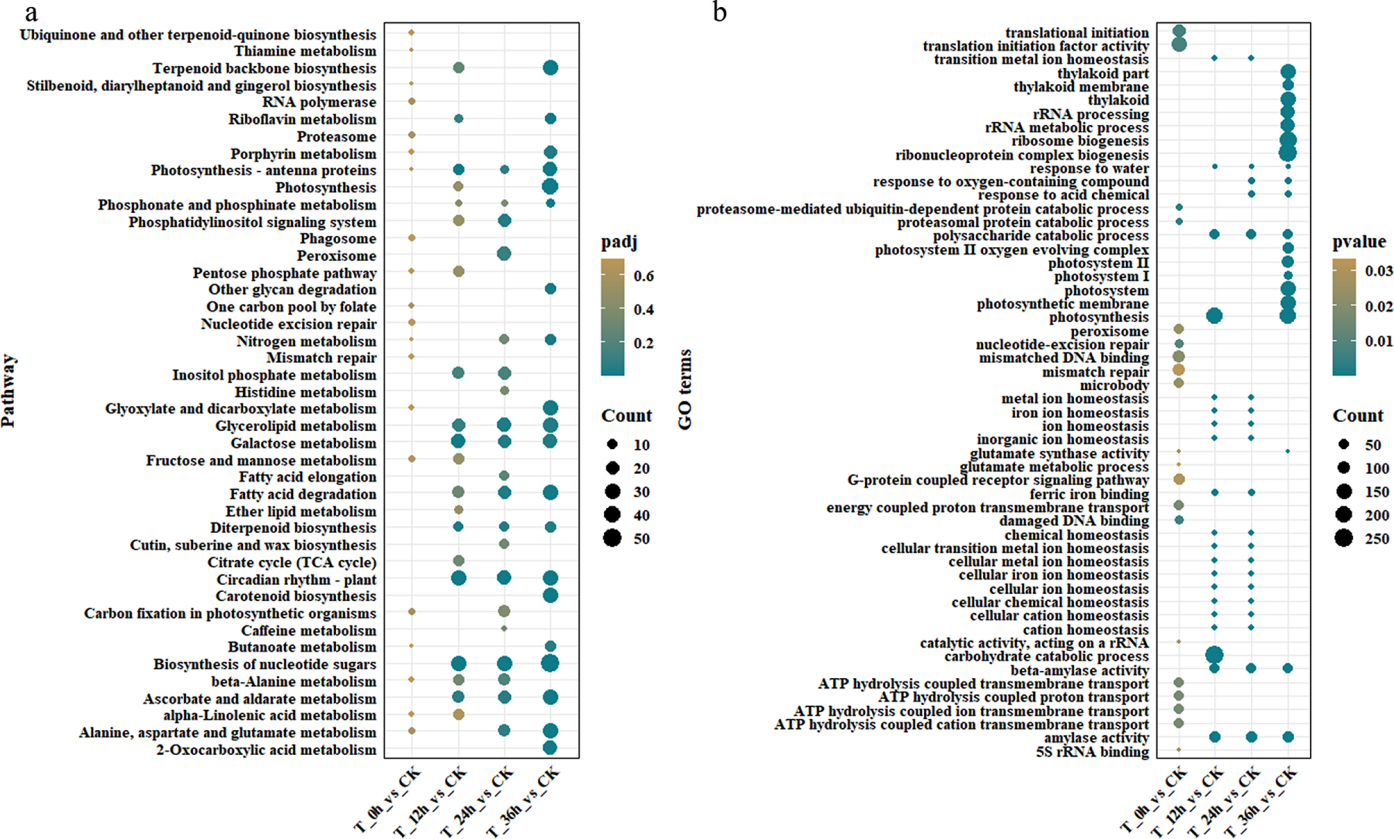
KEGG (**A**) and GO (**B**) enrichment analysis of DEGs involved in the response to cold stress in alfalfa. The color of the points depicts the padj (KEGG) and pvalue (GO) value of the pathway. The size of the points corresponds to the number of enriched genes

The cold acclimation-induced genes were mainly associated with the synthesis of ubiquinone and other terpenoid-quinones; stilbenoid, diarylheptanoid and gingerol biosynthesis; RNA polymerase; carbon fixation in photosynthetic organisms; and alanine, aspartate and glutamate metabolism (Fig. 3a). However, genes encoding photosynthesis-antenna proteins were enriched in the cold treatment group after cold acclimation and after 12 h, 24 h, and 36 h of cold stress (Fig. 3a). These results suggest that antenna proteins may play a pivotal role in the cold stress response in alfalfa. In addition, the phosphonate and phosphinate metabolism, glycerolipid metabolism, galactose metabolism, fatty acid degradation, diterpenoid biosynthesis, circadian rhythm-plant, biosynthesis of nucleotide sugars, and ascorbate and aldarate metabolism pathways were enriched after 12 h, 24 h, and 36 h of cold treatment (Fig. 3a). The GO enrichment analysis showed that genes related to ATP hydrolysis, translation initiation factor activity, and mismatched DNA binding were induced by cold acclimation (Fig. 3b). After 12 h of cold stress, DEGs mainly enriched in the GO terms of photosynthesis and carbohydrate catabolic process (Fig. 3b). However, the photosynthesis and carbohydrate catabolic process related gene were reduced after 24 h of cold stress (Fig. 3b). After 24 h of cold stress, genes related to cellular metal ion homeostasis were enriched (Fig. 3b). Furthermore, after 36 h of cold stress, genes mainly related to the GO terms of photosynthesis, thylakoid part, and ribonucleoprotein complex biogenesis (Fig. 3b).

### STEM analysis of genes involved in the cold response of alfalfa

**Fig. 4:**
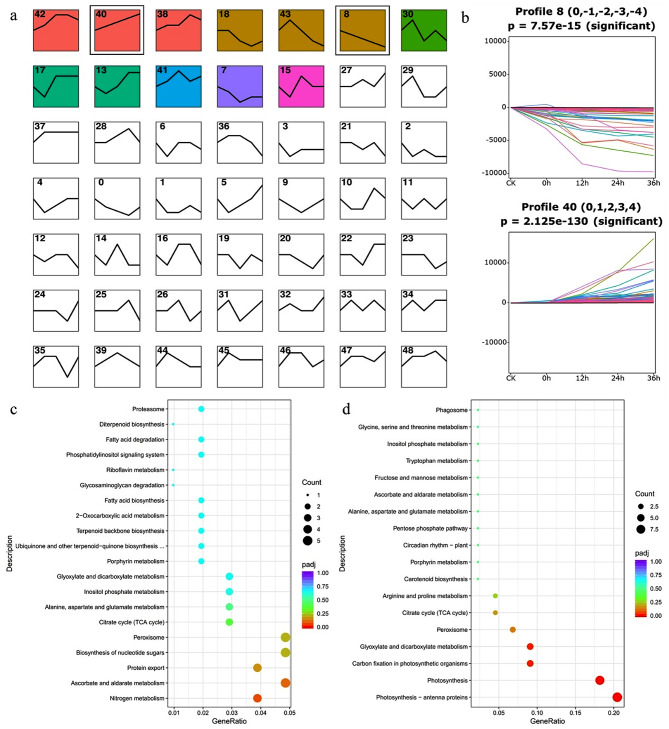
STEM analysis of genes involved in the cold response. (**a**) Significantly enriched gene clusters under cold stress. The colors in the profiles represent the significance of gene cluster enrichment. (**b**) The continuously downregulated (profile 8) and upregulated (profile 40) genes under cold stress. KEGG enrichment analysis of genes in profile 40 (**c**) and profile 8 (**d**). The color of the points depicts the padj value of the pathways. The size of the points corresponds to the number of enriched genes

STEM analysis revealed that 12 clusters were significantly associated with cold stress in alfalfa (Fig. 4a). The gene expression levels in profile 40 increased as the treatment duration increased (Fig. 4b). However, the gene expression levels in profile 8 decreased as the treatment duration increased (Fig. 4b). KEGG enrichment analysis of these genes indicated that the genes within profile 40 were significantly enriched in metabolic pathways such as ascorbate and aldarate metabolism, biosynthesis of nucleotide sugars, peroxisome, nitrogen metabolism, and protein export (Fig. 4c). In contrast, genes within profile 8 exhibited prominent associations with photosynthesis and carbon assimilation mediated by photosynthesis (Fig. 4d). Additionally, the expression levels of eight AP2/ERF transcription factors were consistently upregulated (Table S3). The expression levels of the WRKY, Tify, NAC, HB, HB-BELL, GRAS, C3H, C2H2, C2C2-CO-like, and BBR transcription factors also demonstrated a sustained increase following exposure to cold stress conditions (Table S3). Conversely, the expression levels of GARP-G2-like, C2C2-Dof, bHLH, and MYB-related transcription factors exhibited a consistent decrease in response to cold stress (Table S4). The expression of phytohormone genes was also induced by cold stress. The auxin-responsive proteins SAUR32, SAUR36, and Gibberellin 2-β-dioxygenase 2 and the ABA/WDS-induced proteins were upregulated by cold stress (Table S3).

### Hub genes involved in the response to cold stress in alfalfa

**Fig. 5:**
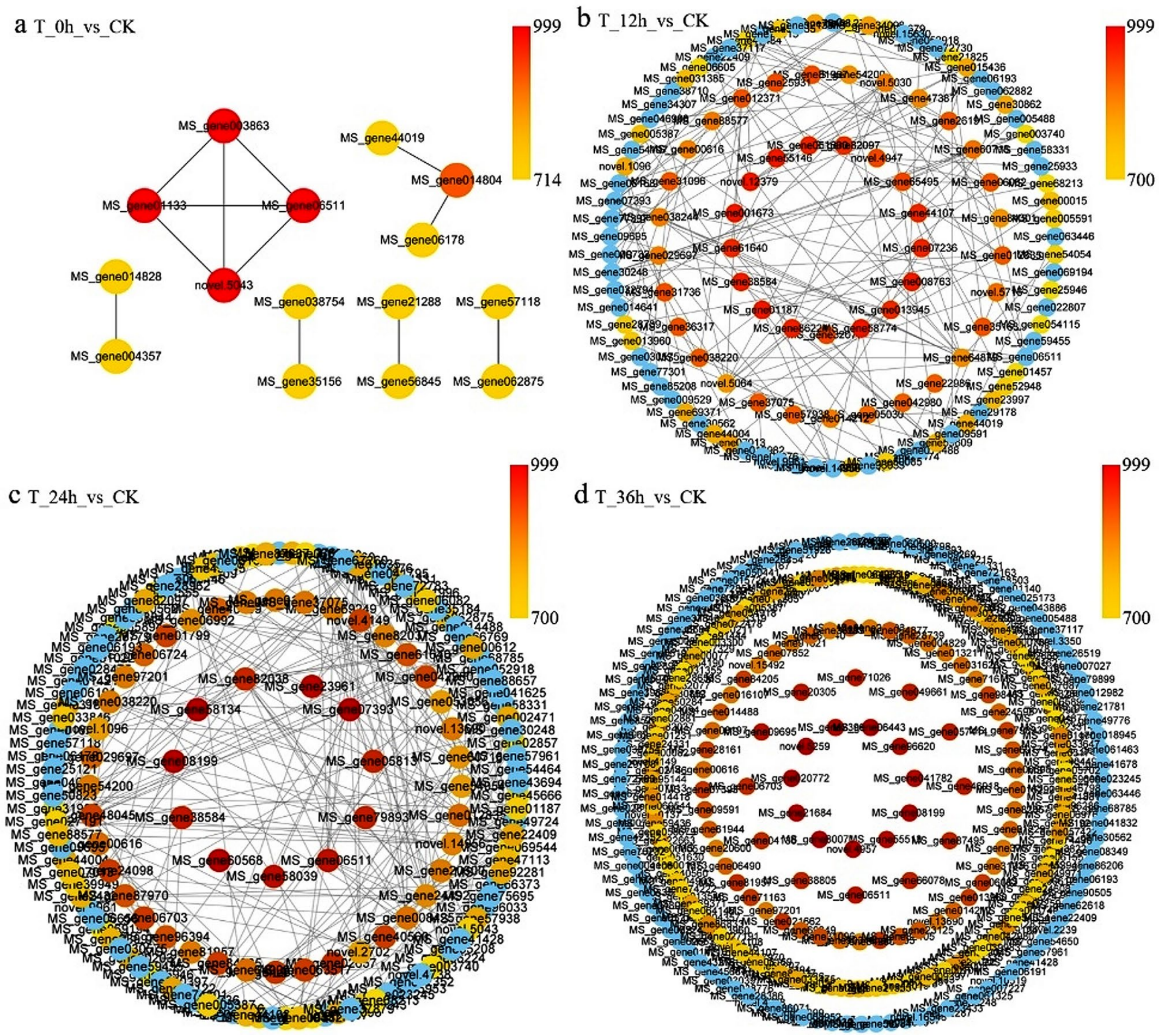
Cold-induced PPI network in alfalfa. Redder colors indicate a higher degree of enrichment

PPI network analysis of DEGs was conducted at 12 h, 24 h, and 36 h after cold acclimation and cold stress. Interactions with scores greater than 700 are depicted graphically (Fig. 5). After cold acclimation, a mere 12 pairs of interactions exhibited a score above 700 (Fig. 5a and Table S5). The expression of ribosomal proteins such as L44, L38, and S26e was initially induced by cold acclimation (Fig. 5a and Table S5). In addition, the expression of the ubiquitin-protein ligase genes E2 and E3, the RNA polymerase II gene Rpb7, the eukaryotic initiation factor 4E, the metallopeptidase family gene M24, and nascent polypeptides was also induced by cold acclimation (Fig. 5a and Table S5). However, after 12 h of cold stress, 174 possible interaction pairs were identified (Fig. 5b and Table S6). The 26 S proteasome non-ATPase regulatory subunit 8 homolog A, ubiquitin-60 S ribosomal protein L40, and 40 S ribosomal protein S2 enriched many downstream genes after cold stress (Fig. 5b and Table S6). After 24 h of cold stress, a total of 246 potential interaction pairs were obtained (Fig. 5c and Table S7). The 40 S ribosomal protein S11, ribosome biogenesis protein BOP1, and 60 S ribosomal protein L10a-1 also enriched numerous downstream genes (Fig. 5c and Table S7). After 36 h of cold stress, the number of pairs of interactions with a score above 700 reached 988 (Fig. 5d and Table S8). Nucleolar protein 14, ANTHESIS POMOTING FACTOR 1, and ribosome biogenesis protein BOP1 were highly enriched after 36 h of cold stress (Fig. 5d and Table S8). These findings indicate that as the duration of stress increases, an increased number of genes participate in the response to cold stress. In addition, ubiquitination-related ribosomal proteins may play a pivotal role in the response to cold stress.

### Plant hormone genes involved in the response to cold stress in alfalfa

**Fig. 6:**
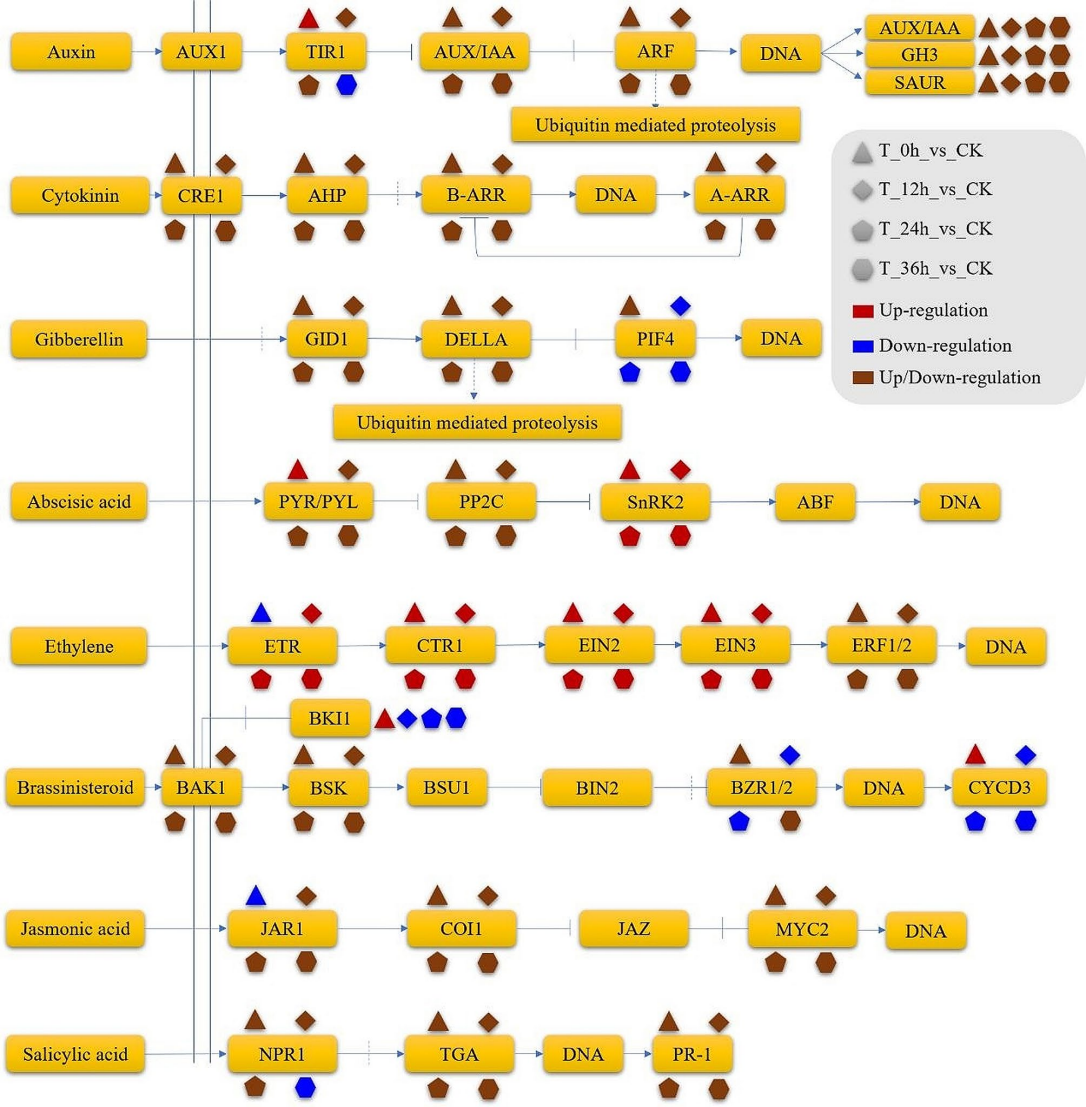
Plant hormone-induced signal transduction under cold stress in alfalfa. The various shapes depicted in the figure indicate distinct comparison groups, with each shape representing a different group. Similarly, the different colors represent genes that were either upregulated or downregulated

Plant hormones play a pivotal role in the defense against cold stress. Therefore, the expression levels of plant hormone genes were further investigated. A total of 550 auxin-related genes, 242 cytokinin-related genes, 104 gibberellin-related genes, 1914 ABA-related genes, 464 ethylene-related genes, 58 BR-related genes, 43 JA-related genes, and 227 salicylic acid-related genes were identified (Tables S9–S15). The expression of several genes involved in auxin signal transduction was significantly affected under cold stress conditions. Specifically, the genes TIR1, AUX/IAA, ARF, GH3, and SAUR exhibited significant upregulation or downregulation (Fig. 6 and Table S9). Additionally, the auxin efflux carrier components PIN3 and PIN4 were notably downregulated after exposure to cold stress (Table S9). Under both cold acclimation and cold stress conditions, the genes NAA15 and NAA25, which encode part of the N-terminal acetyltransferase A complex auxiliary subunit, were upregulated (Table S9). In contrast, genes belonging to the auxin transporter-like protein LAX family were upregulated after cold acclimation but downregulated during prolonged cold stress (Table S9). Moreover, the genes encoding GH3.1, GH3.5, GH3.5, and GH3.10 in the indole-3-acetic acid-amido (IAA) synthetase family were significantly upregulated after cold stress (Table S9). Among the dormancy-associated protein family genes, DRMH3 and DRM1 were also significantly upregulated after cold stress (Table S9). Conversely, the AUXIN SIGNALING F-BOX genes AFB2 and AFB3, which are involved in auxin signaling, were downregulated after cold stress (Table S9).

The genes associated with the transduction of cytokinin signals, namely, CRE1, AHP, B-ARR, and A-ARR, were induced in response to cold stress (Fig. 6 and Table S10). Among them, APRR1 and APRR5, members of the ARR gene family, were upregulated, whereas the transcript levels of APRR2 decreased following cold stress (Table S10). Similarly, after cold stress, the expression of AHP1, a member of the AHP gene family, decreased, whereas the transcript levels of AHP4 and AHP5 increased (Table S10). Moreover, the expression of the cytokinin hydroxylase gene CYP735A2 was upregulated following cold stress, whereas that of the cytokinin dehydrogenase 1 (CKX1) gene was significantly downregulated (Table S10).

Cold stress also regulated genes related to gibberellin. The expression of genes involved in gibberellin signal transduction, specifically GID1, DELLA, and PIF4, was significantly regulated under cold stress (Table S11). The expression of the PIF4 genes was downregulated by low temperatures. Furthermore, cold stress led to upregulation of the transcript levels of the gibberellin receptor GID1B, chitin-inducible gibberellin-responsive protein 1 (CIGR1), and gibberellin 2-beta-dioxygenase 1 (G2OX1). The regulation of these genes may have significant implications for the processes of gibberellin signal transduction and metabolism under cold stress.

In the present study, among the genes related to plant hormones, ABA-related genes exhibited the highest frequency (Table S12). Following cold acclimation, the expression of ABA receptor genes was upregulated. Specifically, the transcript levels of PYL8 were upregulated under cold stress (Table S12). Conversely, only a few transcripts of PYL4 were downregulated after exposure to cold stress. The response to cold stress in alfalfa involved a total of 223 PP2C genes, the majority of which were upregulated. Although these genes displayed relatively minor changes in expression levels during cold acclimation, they exhibited more substantial alterations under continuous cold stress conditions (Table S12). In addition, 143 serine/threonine-protein kinase (SnRK) genes were implicated in the response to cold stress, with the ABA signal transduction gene SnRK2 being upregulated under cold stress conditions (Table S12). These findings suggest that ABA signaling plays a crucial role in conferring cold stress resistance in alfalfa.

In the ethylene signal transduction pathway, the ethylene receptor (ETR) genes ETR1 and ETR2, the serine/threonine-protein kinase CTR1, and the ethylene-insensitive proteins EIN2 and EIN3 were upregulated in response to cold stress (Fig. 6 and Table S13). A total of 176 genes belonging to the ethylene-responsive transcription factor (ERF) family were involved in the cold stress response in alfalfa. Notably, ERF53, ERF61, ERF109, and ERF110 were upregulated under cold stress conditions. ERF17 was upregulated specifically after 36 h of cold stress (Fig. 6 and Table S13). Additionally, the mitogen-activated protein kinases (MAPKs) MAPKKK20 and MAPKKK18 were also upregulated in response to cold stress (Fig. 6 and Table S13).

Most of the 58 BR-related genes were downregulated. Certain transcripts of brassinosteroid-responsive RING protein 1 (RBH1) were upregulated under cold temperature stress (Fig. 6 and Table S14). The expression of genes encoding serine/threonine-protein kinases, such as BSK1, BSK3, and BSK5, increased in response to cold acclimation and cold stress (Table S14). Additionally, cold acclimation induced the upregulation of BKI1, a brassinosteroid kinase inhibitor, as well as Cyclin-D3 genes (CYCD3-3, CYCD3-1). However, during cold stress conditions, genes encoding BKIs and CYCDs were downregulated (Table S14).

Cold acclimation induced the upregulation of JA carboxyl methyltransferase 2 (JMT2), jasmonoyl-L-amino acid synthetases (JAR4, JAR6), coronatine-insensitive protein 1 (COI1), and the transcription factor MYC2 (Fig. 6 and Table S15). Under cold stress, most of these genes were downregulated, but a majority of the MYC2 genes remained upregulated (Fig. 6 and Table S15).

In response to cold stress, the majority of salicylic acid-related genes were downregulated (Fig. 5 and Table S16). However, TGA9, a key transcription factor in the salicylic acid signaling pathway, exhibited significant upregulation under cold stress conditions, while TGA4 and TGA3 were downregulated (Fig. 5 and Table S16). Furthermore, numerous genes encoding pathogenesis-related proteins (PRs) were regulated in response to cold stress (Fig. 5 and Table S16). Genes encoding salicylic acid-binding proteins (SABPs), as well as genes encoding NPR1, a salicylic acid signaling component, were predominantly downregulated. These genes could play a crucial role in mediating the response to cold stress in alfalfa (Fig. 5 and Table S16).

### Transcriptional factors (TFs) involved in responding clod stress in the alfalfa

**Fig. 7:**
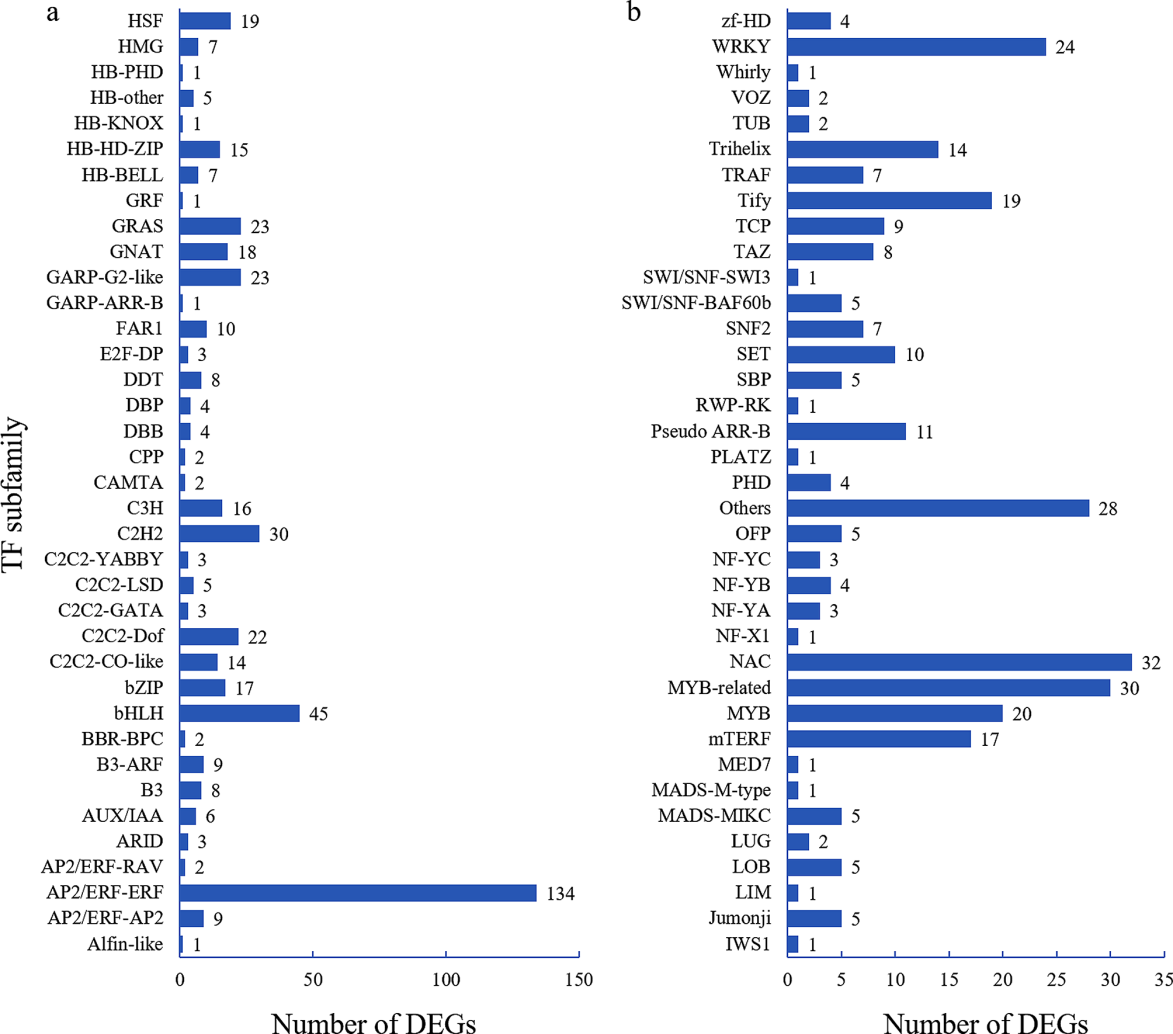
Differentially expressed transcription factors involved in responding cold stress in alfalfa

A total of 783 differentially expressed TFs were identified after cold acclimation and 12 h, 24 h, 36 h of cold stress (Fig. 7). These TFs were further categorized into 74 subfamilies (Fig. 7). The AP2/ERF subfamily comprises the highest number of differentially expressed TFs (Fig. 7a). Moreover, a significant number of differentially expressed TFs from the MYB, NAC, bHLH, C2C2, and WRKY families are involved in responding to cold acclimation and cold stress (Fig. 7). With prolonged cold stress duration, the expression levels of 8 AP2/ERF, 3 GRAS, and 3 Tify TFs were also elevated (Table S3). The abundance of MYB-related, GARP-G2-like, C2C2-Dof, bHLH, and ERF were decreased with the increase of the time of cold stress (Table S4). These TFs may highly correlated with the cold stress responding in alfalfa.

## Discussion

Cold stress affects plants by inhibiting their growth and development, damaging cell membranes, disrupting physiological metabolism, altering hormone regulation, and activating antioxidant systems [4, 32]. The overwintering of alfalfa is typically reduced under extremely cold conditions, which subsequently impacts yield [33]. Investigating the mechanisms of cold resistance in alfalfa, screening cold-tolerant germplasms, and identifying cold tolerance genes are crucial for improving the overwintering survival rate of this crop through genetic modification approaches. This study examined the cold tolerance of the alfalfa variety D, which was developed by our research group. It was observed that Daye NO.3 can successfully overwinter at an altitude of 4270 m on the Qinghai‒Tibet Plateau. Compared with those of the L genotype, better performance and antioxidant capacities were observed for the D genotype under long-term cold stress (Fig. 1). Hence, we investigated the potential underlying molecular mechanisms.

### Photosynthesis genes are regulated by cold stress

Low temperatures decrease the fluidity of cell and chloroplast membranes, resulting in the formation of ice crystals in the cytosol [34]. This damage to membranes interrupts crucial cellular and physiological functions. Additionally, cold stress has negative impacts on both photosynthesis and CO_2_ fixation [35]. Similar to other abiotic stresses, cold stress stimulates the production of genes that encode crucial antioxidants, preserving the photosynthetic process [36]. It is now known that these genes encode the subunits of the chloroplast NADH dehydrogenase-like (NDH) complex, which facilitates the antimycin A-resistant pathway of cyclic electron transport around photosystem I [37]. Cold stress inhibited the expression of the photosynthetic NDH subunit of lumenal location 2, subcomplex B1, and subcomplex B5 (Table S4). This inhibition has the potential to disrupt the electron transfer process in alfalfa and suppress alfalfa photosynthesis [38]. Light reactions and dark reactions are mediated by two complex multiunit reaction centers known as photosystem I (PSI) and photosystem II (PSII). PSI and PSII collaborate to transform solar energy into chemical energy through the facilitation of electron transport across a sequence of electron carriers. Ultimately, this intricate process yields crucial compounds for photosynthesis, namely, NADPH and ATP [39]. The downregulation of photosystem I reaction center subunit N, VI-2, subunit O, and photosystem II 10 kDa polypeptide, a 5 kDa protein, the core complex protein psbY, and the reaction center X protein (PsbX) in alfalfa under cold stress suggested negative effects of cold stress on photosynthesis in alfalfa (Table S4). However, more stay green leaves were observed in the cold tolerant variety (Fig. 1). We noticed that the chlorophyll b reductase NYC1, Chlorophyll A-B binding protein, and photosystem I assembly 2 were continuous up-regulated (Table S3). These genes may contribute to maintain higher abundance of chlorophyll of alfalfa under cold stress [24].

### Upregulation of nitrogen metabolism and TCA cycle genes involved in cold stress defense in alfalfa

The activation of nitrogen metabolism pathways occurs under various abiotic stress conditions, such as drought stress [40], heavy metal stress [41], salt stress [42], and cold stress [43]. The production of nitric oxide (NO) through nitrate reductase (NR) plays a critical role in enhancing freezing tolerance during cold acclimation in *Arabidopsis*. Cold acclimation prompts the upregulation of NR-dependent NO synthesis, leading to increased accumulation of proline (Pro) and improved freezing tolerance. Mutants lacking functional NR display lower NO levels and reduced freezing tolerance, underscoring the importance of NR-dependent NO production in the response to cold acclimation [44]. Exogenous NO application can enhance the cold tolerance of *Triticum aestivum* L [45]. Cold stress can lead to the upregulation of NR gene expression in alfalfa, which may enhance its resistance to low temperatures (Table S3). Glutamine synthetase (GS) is a key enzyme involved in assimilating NH_4_^+^ to synthesize glutamine (Gln). Increased expression of the chloroplast GS gene in rice leads to improved salt tolerance in transgenic plants [46]. Glutamate dehydrogenase (GDH) converts glutamate to α-ketoglutarate, contributing to the accumulation of proline and the generation of energy [47]. Aconitate hydratase is also a key enzyme in the TCA cycle that catalyzes the synthesis of α-ketoglutarate. α-Ketoglutarate, through a series of reactions, is eventually converted to fumaric acid and FADH2 by succinate dehydrogenase (SDH) [48]. Cold stress can lead to the upregulation of genes involved in nitrogen metabolism pathways, such as NR, GS, and GDH, as well as genes associated with the TCA cycle, such as those encoding aconitate hydratases and SDH (Table S3). These results suggest that nitrogen metabolism and the TCA cycle may play a role in defending alfalfa against cold stress [49].

### Transcriptional regulation and hormone signals involved in cold signal transduction in alfalfa

Plant transcription factors play a crucial role in the early transduction of stress-induced signals [50]. Previous studies have demonstrated that the expression of the WRKY transcription factor can be upregulated by exogenous ABA treatment, leading to enhanced cold tolerance in cucumber (*Cucumis sativus* L.). Overexpression of the CsWRKY46 gene in *Arabidopsis* confers improved cold tolerance in transgenic plants [51]. Similar findings have also been validated in investigations of the mechanisms underlying cold tolerance in wheat [52]. The magnesium-protoporphyrin IX chelatase large subunit is considered an ABA receptor that has the ability to directly regulate WRKY transcription factors, further exerting a negative regulatory effect on ABA signaling [53]. This study revealed that the expression of WRKY transcription factors in alfalfa was consistently upregulated under cold stress conditions (Table S3). Similarly, ABA receptor genes were upregulated, and genes involved in the ABA signaling pathway, such as PYL8, PYL4, PP2C, and SnRK2, were found to be involved in the response to cold stress (Table S12). These results suggest that the upregulation of WRKY transcription factors and activation of the ABA signaling pathway may contribute to the enhancement of cold tolerance in alfalfa.

Ethylene and JA typically act synergistically and participate in plant responses to adverse stress conditions [54]. We also observed that the genes ERFs, ETR1, CTR1, EIN2, and EIN3, which are involved in the ethylene signaling pathway, were upregulated under cold stress (Fig. 6, Table S3). The *etr1-1* and *ein3-1* mutants of *Arabidopsis thaliana* exhibited increased tolerance to cold stress [55]. The application of JA can enhance the cold resistance of peach fruit through the regulation of ethylene and sugar metabolism [56]. The JA signaling pathway gene-encoded Jasmonate ZIM-domain protein directly interacts with the ethylene-insensitive protein EIN3 and further regulates plant cold tolerance through the C-repeat binding factor (CBF) or MYC2 gene [57]. In the present study, cold stress also upregulated the expression of the majority of genes involved in the JA signaling pathway, MYC2 (Table S15). These findings suggest that the ethylene and JA signaling pathways may have significant implications for the cold tolerance of alfalfa.

The GRAS transcription factor of alfalfa was continuously upregulated under cold stress (Table S3 and S17). The role of the GRAS domain transcription factor PAT1 in enhancing plant cold tolerance through the regulation of JA synthesis has been supported by prior research [58]. Gibberellins exert their effects at the cellular level by binding to GID1 receptors (GIBBERELLIC ACID INSENSITIVE DWARF1), which subsequently promotes the ubiquitylation and proteasomal degradation of DELLA repressors [59]. PTA and DELLA transcription factors belong to the GRAS subfamily of genes [60]. These results indicate that transcriptional regulation mediated by gibberellin signaling also plays a crucial role in the alfalfa response to cold stress.

### Ubiquitin-related ribosomal proteins and genes that act as hub genes in the PPI network

Ribosomal proteins are highly conserved components of essential cellular organelles and are crucially involved in the translation of mRNA to facilitate protein synthesis. The ribosomal protein L44 (RPL44) from the halophilic fungus *Aspergillus glaucus* enhances abiotic stress resistance in yeast and plants [61]. A study found that deletion of the ribosomal protein L33 has no impact on plant growth under normal conditions but decreases the resistance of plants to cold stress [62]. Zhang et al. [51] proposed that the ribosomal protein S5 is involved in photosynthesis and that the overexpression of this protein in plants leads to increased tolerance to cold stress. The differential expression of the ribosomal proteins L44, L38, and S26e induced by cold acclimation may play a significant role in enhancing the cold tolerance of alfalfa (Fig. 5a and Table S5).

Ubiquitination, through the modulation of regulatory protein levels and activity, plays a pivotal role in regulating the transcriptional changes necessary for adaptation to abiotic stresses [63]. The ubiquitination pathway comprises three key enzymes that sequentially perform their functions during the process. E1 activates ubiquitin molecules and attaches them to ubiquitin. E3 then transfers ubiquitin from E2 to the lysine residues of the substrate protein [63]. The 26 S proteasome recognizes ubiquitinated proteins, and deubiquitinating enzymes (DUBs) eliminate ubiquitin chains from substrate proteins to recycle them. Proteases within the 26 S proteasome subsequently degrade the deubiquitinated proteins [64]. Overexpressing the U-box type E3-ubiquitin ligase *OsPUB2* or *OsPUB3* in rice plants leads to a cold tolerance phenotype characterized by improved survival rates, enhanced chlorophyll content, and reduced ion leakage [65]. The enrichment of 26 S proteasome non-ATPase regulatory subunit 8 homolog A, ubiquitin-60 S ribosomal protein L40, and 40 S ribosomal protein S2 may contribute to enhancing the cold tolerance of alfalfa (Fig. 5b and Table S6).

## Conclusions

Cold acclimation triggered the expression of a subset of cold-responsive genes in response to cold stress, with early participation of ribosomal proteins related to ubiquitination. With increasing duration of cold exposure, an increasing number of genes participate in the response to cold stress. Genes associated with the photosynthetic pathway exhibit consistent expression during cold stress. Genes involved in nitrogen metabolism, the TCA cycle, and hormonal signaling pathways were activated during cold stress. These genes may play a role in alfalfa’s resistance to cold stress. Furthermore, ubiquitination-related ribosomal proteins were enriched in the PPI network, suggesting their potential as central genes in the alfalfa response to cold stress.

### Electronic supplementary material

Below is the link to the electronic supplementary material.

Supplementary Material 1

Supplementary Material 2

Supplementary Material 3

Supplementary Material 4

Supplementary Material 5

Supplementary Material 6

Supplementary Material 7

Supplementary Material 8

Supplementary Material 9

Supplementary Material 10

Supplementary Material 11

Supplementary Material 12

Supplementary Material 13

Supplementary Material 14

Supplementary Material 15

Supplementary Material 16

Supplementary Material 17

Supplementary Material 18

## Data Availability

The datasets generated and/or analysed during the current study are available in the NCBI repository (https://submit.ncbi.nlm.nih.gov/subs/sra/SUB14335695/overview), with the accession No. of PRJNA1091571.
