# Supplementary figures and images for: Transcriptomic analyses provide molecular insight into the cold stress response of cold-tolerant alfalfa
Source: BMC Plant Biol. 2024 Aug 3;24:741. doi: 10.1186/s12870-024-05136-y (PMC11297790; doi:10.1186/s12870-024-05136-y)

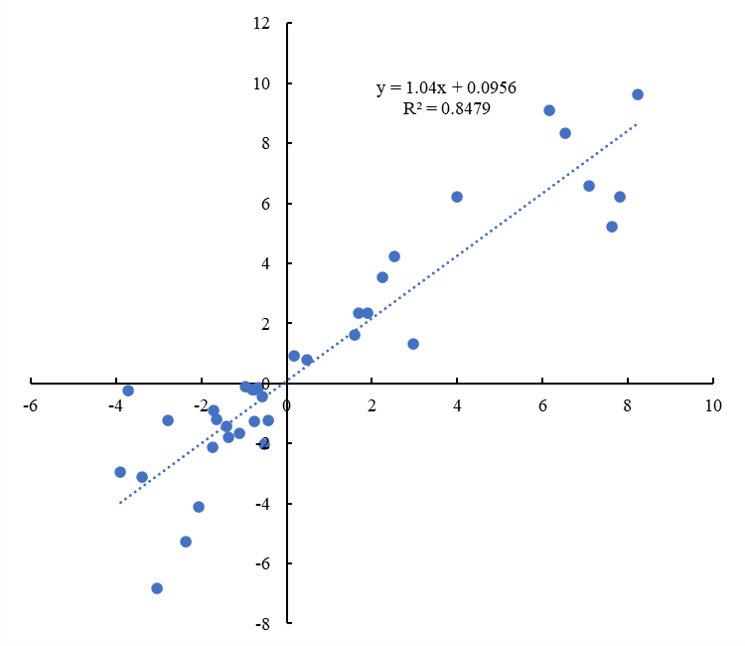

Supplement: Supplementary file 18 — Supplementary Material 18 [file 12870_2024_5136_MOESM18_ESM.tif]
